# Self-Assembly of Functional Nucleic Acid-Based Colorimetric Competition Assay for the Detection of Immunoglobulin E

**DOI:** 10.3390/s19102224

**Published:** 2019-05-14

**Authors:** Xuexia Lin, Caiyun Yu, Honggui Lin, Cui Wang, Jianlong Su, Jie Cheng, Ranjith Kumar Kankala, Shu-Feng Zhou

**Affiliations:** 1 Department of Chemical Engineering & Pharmaceutical Engineering, College of Chemical Engineering, Huaqiao University, Xiamen 361021, China; caiyunYu001@163.com (C.Y.); sujianlong_123@163.com (J.S.); chengj@hqu.edu.cn (J.C.); ranjithkankala@hqu.edu.cn (R.K.K.); 2 School of Marine Engineering, Jimei University, Xiamen 361021 China; linhongui36@163.com; 3 Applied and Environment Microbiology, Department of Biology, Georgie State University, Atlanta, GA 30303, USA; lorna.cui@hotmail.com

**Keywords:** functional nucleic acids, DNAzyme, immunoglobulin E, colorimetric competition detection

## Abstract

In this work, we have developed a simple and rapid colorimetric assay for the detection of immunoglobulin E (IgE) using functional nucleic acids (FNAs) and a solid-phase competition enzyme-linked immunosorbent assay (ELISA). The FNAs including aptamer of recombinant IgE, G-quadruplex and its complementary fragments were immobilized on 96-well microplates to achieve recognition and detection of IgE in biological samples. The G-quadruplex DNAzyme catalyzed 2,2′-Azino-bis (3-ethylbenzothiazoline-6-sulfonic acid (ABTS)-hemin-H_2_O_2_ system was used to improve the sensitivity of colorimetric assay. In the presence of IgE, the hairpin structure and G-quadruplex would be destroyed, resulting in the inactivation of DNAzyme and subsequent reduction of its absorbance. This cost-effective approach detected IgE in the linear range from 5.0 pg/mL to 500 ng/mL, with the limit of detection (LOD) of 2.0 pg/mL, under optimal conditions. Moreover, the developed method was successfully applied to the rapid detection of IgE in human urine, indicating a great potentiality of this approach in clinical diagnosis and other biomedical applications.

## 1. Introduction

Proteins, the natural biomolecules, often serve as biosignaling molecules, molecular indicators, and biomarkers. Proteins have attracted enormous research interest because of their crucial roles in clinical diagnosis, proteomics, and metabolomics. Detection and quantification of proteins during disease prevention and treatment can assist the clinicians in selecting proper treatments and monitoring patient’s responses, adjusting the therapeutic regimens timely and properly. In general, several traditional immunoassays, such as enzyme immunoassay [1], fluoroimmunoassay [2], chemiluminescence immunoassay [3], lateral-flow assay [4], and protein microarrays [5], are commonly used for protein detection. Predominantly, these techniques rely on ELISA and antibody specificity. However, most of these approaches suffer from several shortcomings, such as lack of robustness and variable stability of the employed antigens.

Recently, FNAs, containing nucleic acid aptamer (single-stranded oligonucleotides from DNA/RNA libraries that can bind specific molecular targets) and DNA enzymes (DNAzymes) have become an important tool in protein detection assay [6]. Due to their notable advantages in the detection of proteins, rationally designed FNAs can establish a wide variety of FNA-based assays, such as colorimetric bioassay [7,8], microchip electrophoresis [9], fluorescent [10], and electrochemical [11] and luminescent assay with switch-on probe [12]. Among them, the colorimetric FNA bioassay has been readily developed using solid-phase protein detection, such as microfluidic chip [13], cellulose membrane [14], and paper [15]. Meanwhile, most of the available commercialized technologies for immunoassays have been developed based on microplate technologies. Consequently, there is an opportunity to develop an efficient, rapid and simple protein detection method based on technologies combined with microplate and FNAs for its clinical utilization.

In general, serum and urine are prevalently used to determine the protein concentration in the human body [16]. While blood withdrawal can be highly inconvenient due to the invasive sampling and heavy loss, conversely, urine is more applicable for clinical diagnosis and detection of protein levels. Despite large amounts of non-invasively collected urine samples, they contain only small amounts of protein due to the influence of uric acid and some complex matrices. In order to overcome this problem, the development of an ultrasensitive and high-selective method for the determination of protein in the urine is highly required. In this work, a solid-phase competition ELISA has been developed for protein detection with the improved specificity [17] and robustness [18,19], which provided significant differentiation between positive and negative populations.

DNAzymes are DNA enzymes which can catalyze chemical reactions. G-quadruplex DNAzyme exhibit peroxidase-like activity. Efforts to develop catalytic DNAzyme sensors have been made, which allowed the biocatalytic detection in the hybridization process. In addition, it was reported that DNAzyme was coupled with nanoparticles as a colorimetric sensor for thrombin detection [20]. Moreover, unlike other catalytic beacons, the DNAzyme offers specificity and single base mismatches on analyzing the mutants [21]. DNAzyme-ABTS–hemin–H_2_O_2_ system was considered as an effective strategy for the colorimetric assay for proteins [22,23].

Inspired by these facts and considerations, we hypothesized a simple, cost-effective, rapid, ultrasensitive assay for the detection of critical proteins based on FNAs. In this hypothesis, FNAs including aptamer and DNAzyme sequence can be modified on the surface of microwells and then be used to detect protein via absorbance measurements on a microplate reader. Meanwhile, applying the DNAzyme as a catalytic unit and aptamer can improve the sensitivity and selectivity, respectively.

As a proof-of-concept, IgE has been selected as a model analyte because it is an important indicator related to allergy-mediated disorders, atopic dermatitis, and other immunodeficiency-related diseases. IgE was analyzed by FNA-based fragment on competition law using a microplate reader. Initially, the surface of a micro-well was modified with aptamer exploited to specifically recognize IgE. The FNA fragment was designed with a hairpin structure including IgE aptamer, G-quadruplex sequence and its complementary sequence. The hairpin and G-quadruplex structure would be destroyed in the presence of IgE, leading to the reduction of the absorbance signal, based on G-quadruplex catalyzed ABTS–hemin–H_2_O_2_. As a result, the colorimetric assay was easily achieved. Moreover, the developed method was validated in terms of linearity, sensitivity, repeatability, and accuracy. For clinical applications, the IgE would be successfully detected in a urine sample, with great capability and merit. Thus, we believe that the proposed simple and rapid strategy useful for IgE assay will be promising in disease diagnosis.

## 2. Materials and Methods

### 2.1. Reagents and Materials

Platelet-derived growth factor-BB (PDGF-BB), vascular endothelial growth factor 165 (VEGF_165_), epidermal growth factor (EGF), immunoglobulin G (IgG), immunoglobulin E (IgE), tween-20, ABTS and hemin were purchased from Sigma Co. Ltd. (St. Louis, MO, USA). Bovine serum albumin (BSA) was provided by Xiamen Tagene Biotechnology Co. Ltd. (Xiamen, China). Recombinant IgE Kit was purchased from Immuno Clone Co. Ltd (Beijing, China). Tris (Hydroxymethyl) Aminomethane, NaCl, Na_2_HPO_4_, KH_2_PO_4_, Na_2_CO_3_, KCl and NaHCO_3_ were purchased from Sinopharm Chemical Reagent Co. Ltd. (Beijing, China). The coating solution was prepared by 0.05 mol/L sodium carbonate buffer, with a pH of 9.6. The washing solution was prepared by adding 0.05% tween-20 (v/v) to PBS. The FNA was dissolved in TE buffer (10 mM Tris–HCl, pH = 7.4, 1 mM EDTA). The blocking solution was prepared by adding 1% BSA (w/v) to PBS. The assay solution was prepared by adding 0.15 mol/L NaCl to 0.02 mol/L TE buffer, with a pH of 8.0. Biotin was dispersed in Na-phosphate buffer (0.1 M Na-phosphate, pH = 6.6, 1 mM EDTA, 1 M NaCl). Streptavidin was dissolved in 0.1 M phosphate buffer saline (PBS) buffer (pH = 7.4).

The FNAs were synthesized from Sangon Biotech. Co. Ltd. (Shanghai, China) as following. 5′-GT**G GG**T AGG **GCG** GGT TGG AAG CTT TAA CTC A*GG GGC ACG TTT ATC CGT CCC TCC TAG TGG CGT GC***C CC**G CGC-biotin-3′. The underlined fragments refer to the first complementary sequences to the fragment (GCG) in G-quandruplex. The italicized fragment was the aptamer of IgE and the dissociation constants (Kd) of 10 nM aptamer ranged from 0.01 nM to 0.3 nM [23,24]. The bold and underlined fragment was the second complementary fragment which is complement to the sequences (GGG) in G-quandruplex and is the partial sequences to IgE aptamer.

### 2.2. Sample Preparation

A protein stock solution was prepared by dispersing IgE at a concentration of 50 μg/mL in the assay solution, stored at −20 °C. The working solution of IgE was prepared in assay buffer. Other proteins were also prepared by dispersing them respectively in the assay buffer. Urine samples were donated by group members and were centrifuged at 1000 g for 10 min to remove the deposition immediately. After centrifugation, the urine samples were stored at −20 °C. Before the experiment, the urine temperature was restored to room temperature.

### 2.3. Immobilization of Biotinylated FNAs on Microplate

The captured aptamer was immobilized in the microwell through the streptavidin-biotin technique by following steps. Briefly, the biotinylated microwell was washed three times using ultrapure water, and then the microwell was incubated by adding various concentrations of streptavidin in PBS buffer for 30 min in the dark, washed with PBS containing 0.1% SDS and water in sequence and then allowed to air dry. Various concentrations of biotinylated FNAs from 5 to 2500 fmol/µL were suspended in TE buffer and injected into microwells at different concentrations. Furthermore, the coating was performed by depositing 200 µL of biotinylated FNA solutions in each of the microwells. After evaporation, biotin solution (200 µL, 50 µM) was injected into microwell for 15 min to fill the unoccupied biotin binding sites. Non-specifically bound FNAs were removed by three 15-min washes in Na-phosphate buffer at 37 °C. A standard curve was determined by coating similar solutions in additional microwells but without washing.

### 2.4. Procedure for Protein Detection

Initially, 100 μL of various concentrations of IgE solutions were added into corresponding microwels and incubated at 37 °C for 5 min. After the reaction, the wells were washed three times using the washing solution (200 μL). Subsequently, 100 μL of hemin (100 μM) was injected into the microwell, followed by adding 100 μL of ABTS and H_2_O_2_ mixture. After 10 min of incubation at room temperature, the corresponding absorbance values were detected using the microplate recorder. It should be noted that the prepared microplates and all the immunoreagents must be equilibrated to room temperature before the assay for each analysis.

### 2.5. Data Analysis

The assay solution and samples were run in wells, and the absorbance was measured. Calibration curves were obtained by plotting Y against the logarithm of IgE concentration and fitted to the linear equation of Y-logX, where Y = B − B_0_, B_0_ was the absorbance of the assay solution, B was the absorbance of samples, and X was the IgE concentration.

## 3. Results and Discussion

**Figure 1:**
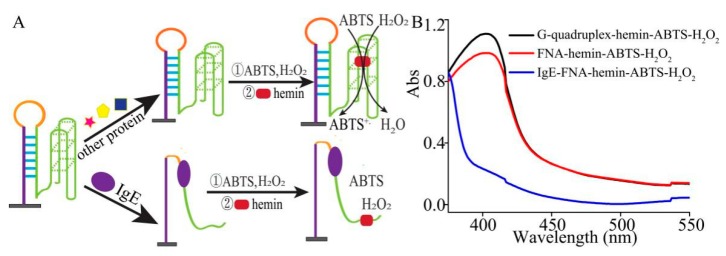
(**A**) Schematic illustration of IgE assay based on functional nucleic acid (FNA); (**B**) UV−vis spectra in the DNAzyme/FNA-mediated ABTS-H_2_O_2_ system.

In this work, we hypothesized that the formation of a stable hairpin structure based on the aptamer of IgE, G-quadruplex and its partial complementary sequence could be efficiently used for detection of protein (Figure 1A). The hairpin and G-quadruplex structure would be formed in the absence of IgE. The hemin would subsequently bind to G-quadruplex and catalyze the reaction with ABTS and hydrogen peroxide to generate a visible color change or absorbance. In the presence of IgE, the hairpin structure was opened and the second complementary fragment was released [23,24]. Then, the first and second complementary fragments can react with G-quadruplex to form a short double chain DNA (dsDNA). Therefore, G-quadruplex would be destroyed. The generation system of absorbance was destroyed, resulting in the reduction of absorbance. The efficiency of the designed FNA was studied (Figure 1B). It was found that in the absence of IgE, the absorbance signals of PS2.M (G-quadruplex), ABTS, hemin, and H_2_O_2_ were strong. On the other hand, in the presence of IgE at 50 pg/mL, the absorbance values of FNAs were significantly low, demonstrating G-quadruplex structures were destroyed. These results indicated that the proposal is feasible, and the designed FNAs include G-quadruplex fragments that can be effectively used for the determination of IgE.

### 3.1. Optimization of Immobilized Streptavidin and FNAs Conditions

**Figure 2:**
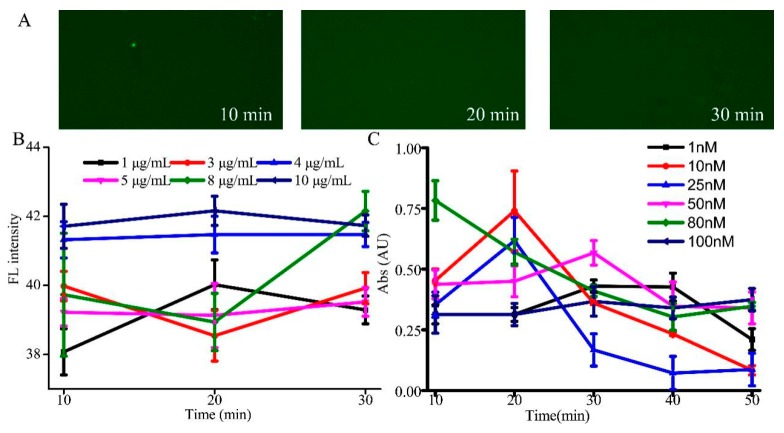
(**A**) Fluorescent images of 10 µg/mL SA coating on the microwell with different time; (**B**) Optimization of streptavidin coating time; (**C**) Optimization of FNAs conditions.

In this strategy, the hairpin structures, FNAs were fixed on the surface of microwell by streptavidin-biotin to capture the target IgE. First, the binding properties of the coat streptavidin into biotin coating microplate were investigated. It was evident that streptavidin can interact with the biotin modified microwell, leaving two additional free sites to bind biotinylated FNAs [25,26]. Indeed, a strong absorbance was observed at the incubation time of 20 min when the concentration of streptavidin was 10 µg/mL (Figure 2B). Furthermore, in order to study the immobilization on the surface of microwell, 10 µg/mL of fluorescently labeled (FITC) streptavidin in PBS was incubated for different time periods (Figure 2A). The FITC-streptavidin coating process was captured by focusing them under a fluorescence microscope with an air-cooled back fin illuminated CCD camera. In contrast, deprived absorbance values were observed with other coating conditions. Therefore, 10 µg/mL and 20 min were taken as the optimum fixed streptavidin conditions for further experiments.

We further optimized the immobilized conditions of FNAs in microwells. It should be noted that the appropriately fixed FNAs conditions are important for the sensitivity and IgE homogeneity assay, as well as the time of incubation. We investigated the binding characteristics of FNA inside streptavidin-coated microwells in Figure 2C. The absorbance was recorded in the presence of different concentrations of the aptamer from 1 to 100 nM at different incubation periods (10 min to 50 min). It can be seen that there is a high value at 20 min when the concentration of FNAs is 10 nM. The changes in the absorbance values would be due to weak non-specific FNAs attachment. Therefore, 10 nM and 20 min were selected as the optimum fixed FNA conditions.

### 3.2. Optimization of the Catalytic System

**Figure 3:**
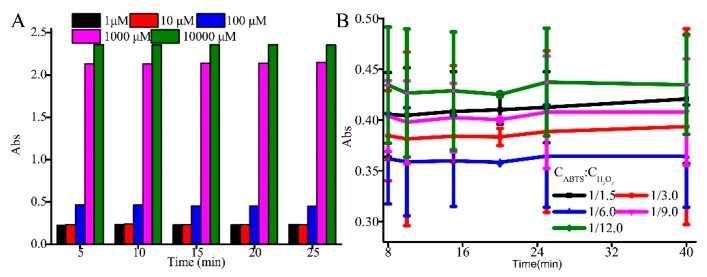
Optimization of the catalytic system (**A**) hemin and (**B**) ABTS-H_2_O_2_.

Indeed, the catalytic conditions of G-quadruplex affect not only the detection time but also the detection accuracy. In addition to G-quadruplex DNAzyme, the high concentration of hemin can accelerate the reaction rate of hydrogen peroxide (H_2_O_2_) and ABTS. Therefore, the high concentration of hemin will lead to a high background value and may eventually affect the accuracy of the analysis. However, the low concentration of hemin could not reflect the change of the concentration in time and extend the analysis time. Figure 3A shows that 100 μM hemin is the most suitable concentration because it has a low background value and relatively rapid detection.

Further, we explored the color signal produced by our system. It was evident that the color signal was also produced dependent upon the concentration ratio of chromogenic reagents including hydrogen peroxide and ABTS. Hydrogen peroxide-mediated oxidation of ABTS generated ABTS^+^ free-radical cation with blue-green color with maximal absorption at 420 nm, which enabled to quantitatively determine the peroxidase-like activity of hemin−DNAzyme complex. As shown in Figure 3B, the G-quadruplex DNAzyme that was complexed with hemin rapidly produced a blue-green absorbance signal at 420 nm with the addition of ABTS and H_2_O_2_ for 25 min. According to the reaction mechanism, the lowest value illustrates effective catalytic efficiency, but inhibition by IgE (Figure 3B). Thus, the ratio of 1:6 of ABTS to hydrogen (C_ABTS_:C_H2O2_ = 1.8 mM:10.8 mM) was chosen as the optimum concentration ratio for the assay.

We further studied the effect of produced color signal with time as the time of chromogenic reaction could affect the absorbance and the detection time. We recorded the reaction time of DNAzyme-ABTS-H_2_O_2_-hemin and the results were plotted in Figure 3B. It was evident that there was an absorbance plateau ranging from 10 to 25 min, illustrating an equilibrium of H_2_O_2_-ABTS and hemin-DNAzyme catalysis. Therefore, the reaction time of 10 min was considered as the shorter analysis time.

### 3.3. Specificity and Sensitivity

**Figure 4:**
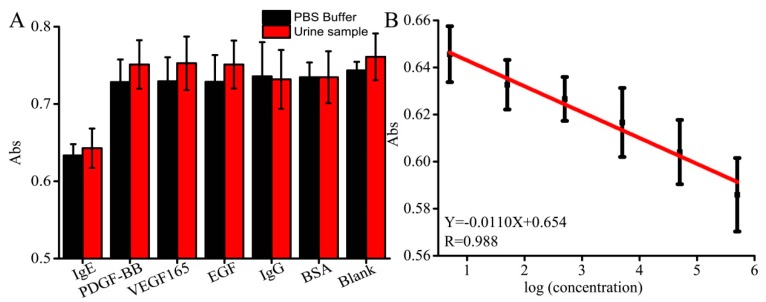
(**A**) Selectivity analysis of FNA-based IgE assay; (**B**) The linear range of the system from 5.0 pg/mL to 5.0 × 10^5^ pg/mL. Every assay was done three times in parallel.

More often, the selectivity, as well as specificity, is considered as the most important factor for effective protein analysis. To investigate the selectivity of the developed system, five other proteins including PDGF-BB, VEGF_165_, EGF, IgG, and BSA were spiked into PBS buffer with a concentration of 500 pg/mL. The blank was used as a control, which is a PBS buffer without any protein. The concentration of other proteins (500.0 pg/mL) was 10-folds higher than of that of IgE (50.0 pg/mL). The absorbance by the non-specific binding was much higher than that of the absorbance by IgE, demonstrating that the developed system resulted in high selectivity towards IgE in Figure 4A. Moreover, the urine sample which IgE was lower than LODwas used to replace PBS solution in order to better mimic the physiological condition. Six kinds of proteins were spiked into the same urine sample, respectively. Figure 4A shows that the absorbance of the spiked PDGF-BB, VEGF, EGF, IgG, and BSA has shown no significant reduction in the signal. These results show that the proposed FNAs exhibited excellent selectivity, indicating their potential application in complex sample analysis. However, the complex sample matrix led to high absorbance. This also indicates that there is very little cross-reaction among proteins or sample matrix. The commercial ELISA IgE kit was used to further study the selectivity of the system, the results show that the proposed system of selectivity is comparable to or even better than that of the commercial ELISA IgE kit.

The absorbance of IgE was further considered to explore the quantitative analysis at different concentrations (5.0, 50, 500, 5.0 × 10^3^, 5.0 × 10^4^, 5.0 × 10^5^ pg/mL). As shown in Figure 4B, there was a good linear correlation of Y = -0.0110X+0.654 (R^2^ = 0.988) in the range from 5.0 to 5.0 × 10^5^ pg/mL with low relative standard deviations (RSDs) between 1.84% and 2.67% (n = 3). We deduced the main reasons for different RSDs would be the roughness of the microporous surface and the deviation of the coating process. By the calculated signal to noise ratio of the, the limit of detection value was around 2.0 pg/mL (~10.5 pM, the molecular IgE is about 190 KD). The sensitivity of the proposed method is comparable to that of the ELISA assay and other methods, such as luminescent, fluorescent, and electrochemical techniques in Table 1. These results demonstrate that not only the FNAs could be used as IgE detection probe, but also that the DNAzyme was effectively a catalytic unit for the ultrasensitive detection of IgE at high sensitivity.

### 3.4. Real Sample Analysis and Recovery

To assess the accuracy and performance of the established method in terms of recovery, various concentrations of IgE were spiked into urine sample by the standard addition method and were determined by the established method (Table 2). The concentrations of IgE obtained from urine sample were in the range from 93.3% to 97.6% with the RSDs between 0.32% and 4.18% (n = 3), indicating that the established method could be applied for analyzing IgE in a urine sample.

To evaluate the efficiency of the developed assay, eight urine samples derived from different people were collected for testing. The treatments and detection strategies were performed according to the developed method and procedure. Every urine sample was detected three times, and the results were shown in Table 2. IgE was detected in sample 2 and sample 4. Their concentrations were in the range from 12 pg/mL to 70 pg/mL, and from 430 pg/mL to 672 pg/mL, respectively. Most importantly, the coefficient of variation was in the range from 2.86 to 9.02% (n = 3), indicating its excellent accuracy. Furthermore, these results are similar to those of commercial kits, demonstrating that the established method is feasible in analyzing clinical samples effectively.

## 4. Conclusions

In this work, an ultrasensitive, highly selective, and rapid method for the determination of IgE in human urine was developed using FNA-based competition law. The proposed method has shown exceptional performance which could be summarized as follows: (i) The usage of FNAs instead of antibodies to immobilize the surface of microwells in this work, which can be achieved target capture and detection.These could not only reduce the analysis procedure but also reduce the cost-effective, making IgE detection simple and fast, (ii) the utilization of microplate reader without changing or updating the instrument made the detection user-friendly and straightforward, (iii) the utilization of competition law and DNAzyme catalyzed ABTS-hemin-H_2_O_2_ system greatly improved the sensitivity, making the colorimetric assay convenient in application for analysis. Finally, the developed method was successfully used in the determination of IgE from human urine, demonstrating that the proposal could be an alternative to ELISA for monitoring IgE in human urine.

## Figures and Tables

**Table 1:** Summary of other methods in recent years to detect IgE.

Assay	Indicator or Amplication	Linearity Range	LOD	Ref.
Luminescence	carbon nanoparticles	0.5 ng/mL to 80 ng/mL	0.2 ng/mL	[27]
Microplate array	silver nanoparticles	20 ng/mL to 320 ng/mL	20 ng/mL	[28]
Electrochemiluminescence	Aptamer-based	9.75 ng/mL to 585 ng/mL	1.56 ng/mL	[29]
Chemiluminescence	Aptamer-based	4.8 pg/mL to 48.79 ng/mL	1.48 pg/mL	[30]
Electrochemical	Aptamer-based	19.4 ng/mL to 19.4 × 10^3^ ng/mL	11.7 ng/mL	[31]
Colorimetric	Aptamer-based and	1.94 × 10^2^ ng/mL to 4.85×10^3^ ng/mL	0.2 nM	[32]
Field effect transistor	gold nanoparticles graphene	9.94 ng/mL to 48.7 ng/mL	9.16 ng/mL	[33]
Electrochemical	aptamer and peptide	0.1 pg/mL to 10 pg/mL	8.19 fg/mL	[34]
Sandwich assay	Enzyme-linked aptamer	0 ng/mL to 3.92 × 10 ng/mL	1.17 ng/mL	[35]

**Table 2:** IgE from human urine sample analyzed by the developed method.

No.	Average Conc. (pg/mL) (n = 3)	Conc. (pg/mL)	SD (pg/mL)	CV (%)	Recovery (%) 50 pg/mL IgE
1	N/A	N/A	N/A	9.02	93.3
2	41	12~70	3	3.09	97.6
3	N/A	N/A	N/A	6.40	96.8
4	620	430~672	160	3.38	97.2
5	N/A	N/A	N/A	3.74	95.4
6	N/A	N/A	N/A	3.14	96.6
7	N/A	N/A	N/A	2.86	94.4
8	N/A	N/A	N/A	4.13	94.5
